# Supplementary material for: The preRC protein ORCA organizes heterochromatin by assembling histone H3 lysine 9 methyltransferases on chromatin
Source: eLife. 2015 Apr 29;4:e06496. doi: 10.7554/eLife.06496 (PMC4442312; doi:10.7554/eLife.06496)
Supplement: Supplementary file 2. — Primers used for validation of ChIP-seq. Our attempts on H3K9me2 ChIP-seq did not succeed because of the technical challenge associated with sequencing the broad H3K9me2 peaks. Similar problems with H3K9me2 ChIP-seq have been previously reported by other studies (Yuan et al., 2009). As an alternate, regions that showed significant reduction of H3K9me3 in the ChIP-seq experiment (as evident by the wiggle plots; Figure 5Da–Dd and Figure 5—figure supplement 1Ba,Bb) were chosen for H3K9me2 ChIP-qPCR validation (Supplementary file 2). DOI: http://dx.doi.org/10.7554/eLife.06496.020 [file elife06496s002.docx]

**Table 1:**

| Primer | Sequence | Chromosome | Start site | End site |
| --- | --- | --- | --- | --- |
| PRKAR1B Fp | TGACAGCATCTGTAACTAGGCTGAA | chr7 | 750583 | 751303 |
| PRKAR1B Rp | CCAGGTGTTTCGCTAGCTTGT |  |  |  |
| CELSR3 Fp | GGTAGCCCTCCTCCACATTCT | chr3 | 48698344 | 48698874 |
| CELSR3 Rp | GGTTTTTGAGCAAGCGCAGTA |  |  |  |
| ZNF536 Fp | CATGGTGTGGGTTCCCTCTT | chr19 | 30867494 | 30868041 |
| ZNF536 Rp | GGGAATACTGGCTGGGTAGTGT |  |  |  |
| ZNF140 Fp | TCCCCAAAACCTCTCCTACGT | chr12 | 133654628 | 133655156 |
| ZNF140 Rp | CGCCTCTACTCCCAGCATTG |  |  |  |
| FAM20A Fp | GCGGCTGCAATAGAAACTTTTT | chr17 | 66596137 | 66596608 |
| FAM20A Rp | CGAACCCCACCAGCTGTT |  |  |  |
| ZNF12 Fp | TCCCCATCCCAGCTTCAG | chr7 | 6742641 | 6743081 |
| ZNF12 Rp | TTGCGTGGCTCCCAGAGT |  |  |  |
| TAF7 Fp | TGGGTCTCATCTTCATCCTCACT | chr5 | 140698820 | 140699099 |
| TAF7 Rp | CATGATGAGCTTCGGGAGATATT |  |  |  |
| PRKG1 Fp | CATGGTGTGGGTTCCCTCTT | chr19 | 30867494 | 30868041 |
| PRKG1 Rp | GGGAATACTGGCTGGGTAGTGT |  |  |  |
